# Supplementary material for: Phase diagrams guide synthesis of highly ordered intermetallic electrocatalysts: separating alloying and ordering stages
Source: Nat Commun. 2022 Dec 10;13:7654. doi: 10.1038/s41467-022-35457-1 (PMC9741640; doi:10.1038/s41467-022-35457-1)
Supplement: Supplementary file 1 — Supplementary Information [file 41467_2022_35457_MOESM1_ESM.pdf]

**Supplementary Information**

# Phase diagrams guide synthesis of highly ordered intermetallic electrocatalysts: separating alloying and ordering stages

**Zeng et al.**

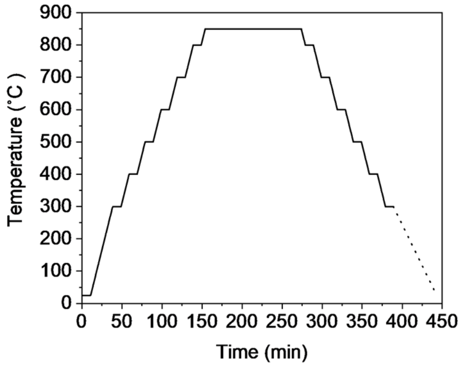


**Supplementary Fig. 1.** In-situ HT-XRD annealing programs. The dash lines indicate naturally cooling stages. The details of annealing program are described in the Method section.

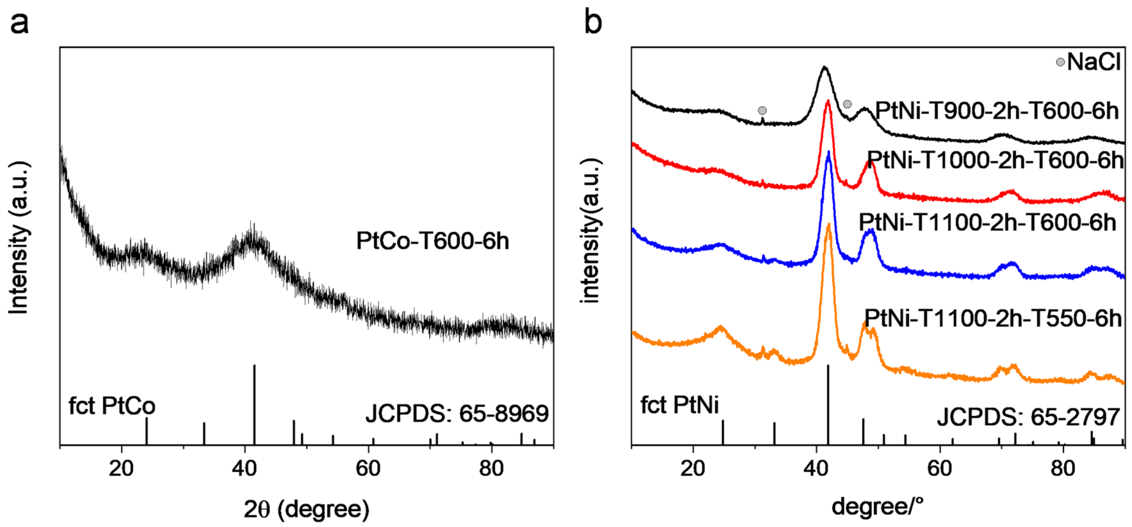


**Supplementary Fig. 2.** XRD patterns of the Pt-Co (a) and Pt-Ni (b) catalysts.

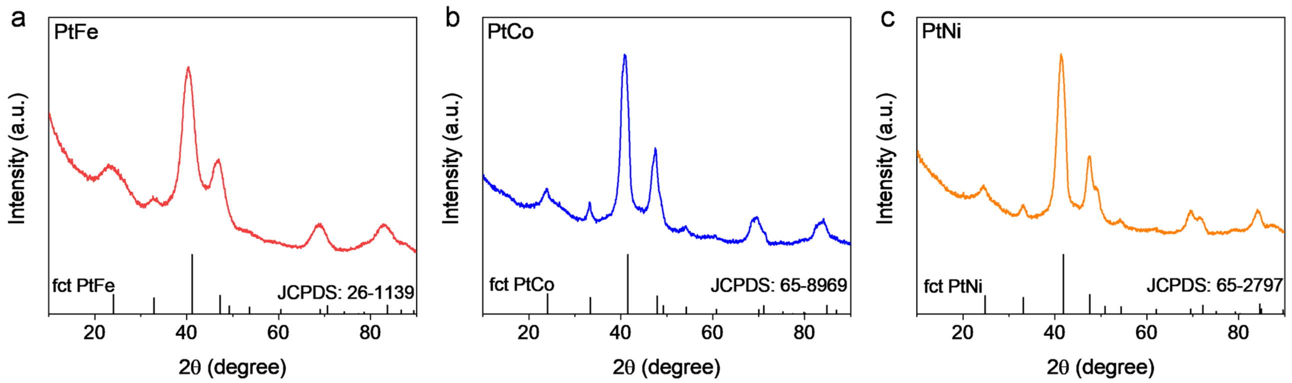


**Supplementary Fig. 3.** (a)–(c) XRD patterns of the PtFe, PtCo, and PtNi catalysts after leaching and $H_2$ annealing treatments, respectively.

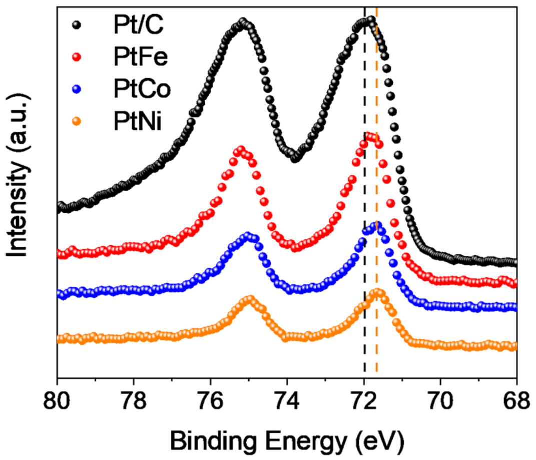

**Supplementary Fig. 4.** XPS spectra (Pt 4*f*) of Pt/C, PtFe, PtCo, and PtNi catalysts.

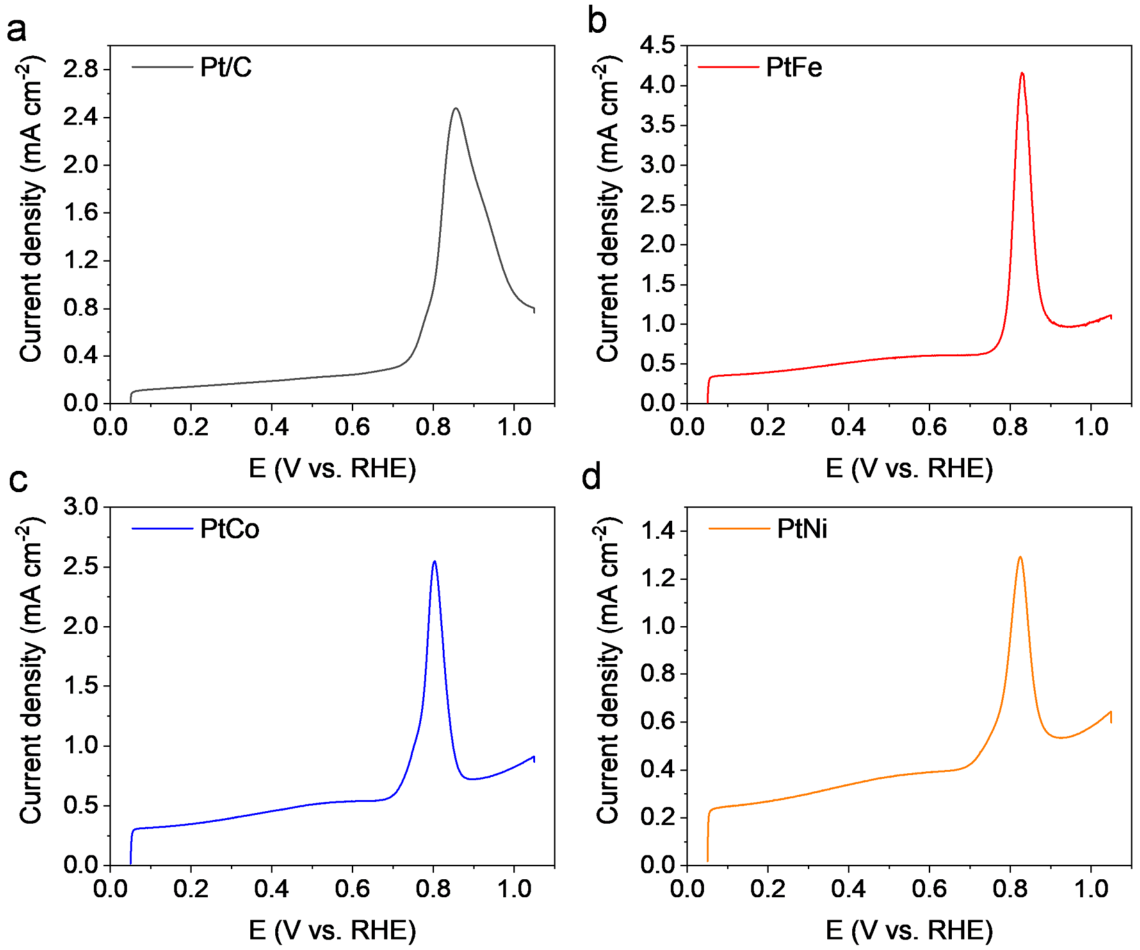


**Supplementary Fig. 5.** CO stripping curves of the Pt/C, PtFe, PtCo and PtNi catalysts in $N_2$-saturated 0.1 M $HClO_4$ with a scan rate of 50 mV $s^{-1}$. CO was adsorbed from CO-saturated 0.1 M $HClO_4$ for 10 min at 0.05 V.

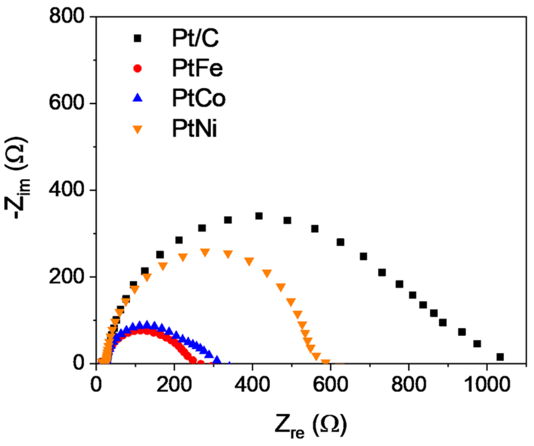


**Supplementary Fig. 6.** Nyquist plots tested at 0.9V (vs. RHE) of Pt/C, PtFe, PtCo, and PtNi catalysts.

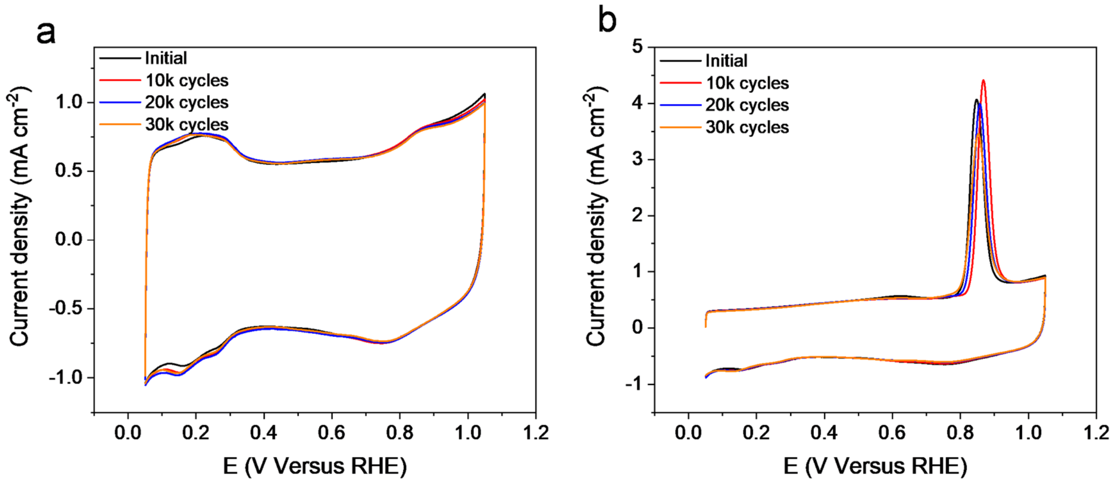


**Supplementary Fig. 7.** CV and CO stripping cures of the PtFe catalyst before and after 30,000 cycles' ADT. These experiments were performed in $N_2$-saturated 0.1 M $HClO_4$ with a scan rate of 50 mV $s^{-1}$. CO was adsorbed from CO-saturated 0.1 M $HClO_4$ for 10 min at 0.05 V.

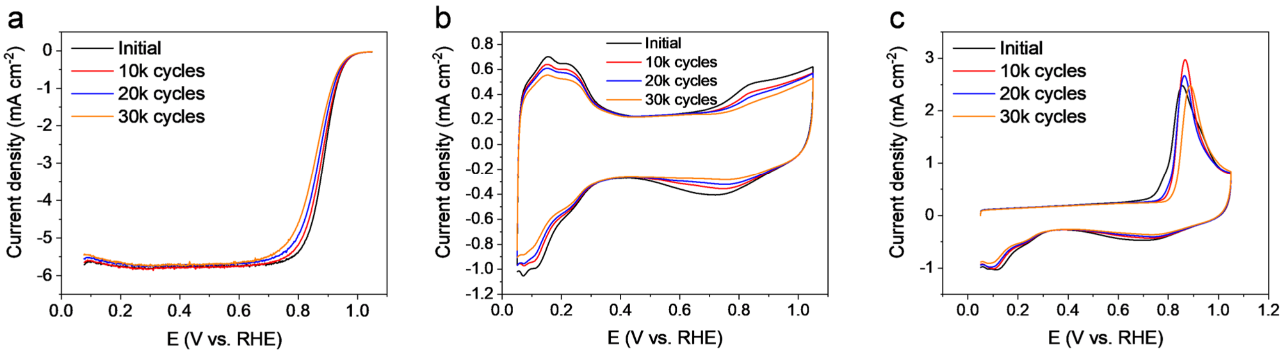


**Supplementary Fig. 8.** a) LSV curves of the Pt/C catalyst before and after 30,000 cycles ADT in $O_2$-saturated 0.1 M $HClO_4$ at a rotation speed of 1600 r.p.m with a scan rate of 10 mV $s^{-1}$. b) CV curves of the Pt/C catalyst before and after 30,000 cycles ADT in $N_2$-saturated 0.1 M $HClO_4$ with a scan rate of 50 mV $s^{-1}$. c) CO stripping curves of the Pt/C catalyst before and after 30,000 cycles ADT in $N_2$-saturated 0.1 M $HClO_4$ with a scan rate of 50 mV $s^{-1}$. CO was adsorbed from CO-saturated 0.1 M $HClO_4$ for 10 min at 0.05 V.

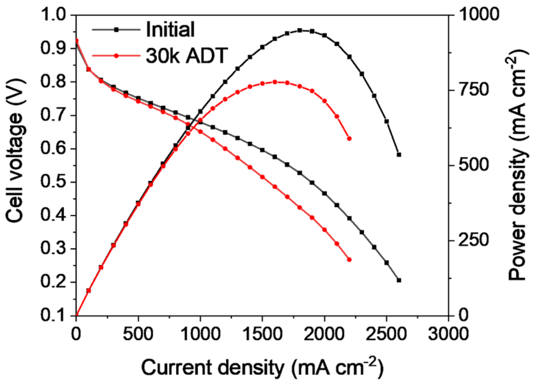


**Supplementary Fig. 9.** $H_2$-air single cell polarization curves of the PtFe cathode before and after ADT. Test conditions: 80 °C, 100% relative humidity, 150 $kPa_{abs}$ $H_2$/air, a fixed gas flow rate of 0.5/2 L $min^{-1}$ for $H_2$/air.

**Supplementary Table 1.** Summary of annealing program, crystal size, average particle size and ordering degree of the Pt-Fe, Pt-Co, and Pt-Ni IMCs catalysts, respectively. The optimal catalysts were marked in red. The (111) interplane distance of the prepared catalysts was calculated by the Bragg's law based on the XRD results.

| Sample | Annealing program | XRD size (nm) | STEM size (nm) | Ordering degree (%) | (111) interplane distance (Å) |
|---|---|---|---|---|---|
| PtFe-T900-2h | 900 ˚C/2h | 2.2 | / | 22 | 2.23 |
| PtFe-T900-2h-T600-6h | 900 ˚C/2h + 600 ˚C/6h | 2.3 | / | 46 | 2.22 |
| PtFe-T1000-2h | 1000 ˚C/2h | 4.1 | / | 56 | 2.21 |
| **PtFe-T900-6h** | **900 ˚C/6h** | **3.1** | **3.5** | **61** | **2.21** |
| PtCo-T600-6h | 600 ˚C/6h | 1.5 | / | / | 2.21 |
| PtCo-T900-2h | 900 ˚C/2h | 2.9 | / | 15 | 2.17 |
| PtCo-T900-6h | 900 ˚C/6h | 3.5 | / | 28 | 2.16 |
| PtCo-T1000-2h | 1000 ˚C/2h | 4.1 | / | 33 | 2.16 |
| **PtCo-T1000-2h-SC** | **1000 ˚C/2h + (-1.1) ˚C/min to 600 ˚C** | **4.2** | **4.6** | **63** | **2.18** |
| PtNi-T900-2h | 900 ˚C/2h | 2.7 | / | / | 2.18 |
| PtNi-T900-2h-T600-6h | 900 ˚C/2h + 600 ˚C/6h | 2.8 | / | / | 2.18 |
| PtNi-T1000-2h | 1000 ˚C/2h | 3.5 | / | / | 2.17 |
| PtNi-T1000-2h-T600-6h | 1000 ˚C/2h + 600 ˚C/6h | 4.2 | / | / | 2.16 |
| PtNi-T1100-2h | 1100 ˚C/2h | 4.2 | / | / | 2.16 |
| PtNi-T1100-2h-T600-6h | 1100 ˚C/2h + 600 ˚C/6h | 4.4 | / | 16 | 2.15 |
| PtNi-T1100-2h-T550-6h | 1100 ˚C/2h + 550 ˚C/6h | 4.1 | / | 28 | 2.16 |
| **PtNi-T1100-2h-T550-12h** | **1100 ˚C/2h + 550 ˚C/12h** | **4.7** | **5.0** | **41** | **2.15** |

**Supplementary Table 2.** Summaries of metal contents and ORR performance of the Pt/C, PtFe, PtCo, and PtNi catalysts. ECSA was measured by CO stripping.

| Catalysts | Pt (wt%) | M/Pt ratio before leaching (atom) | M/Pt ratio after leaching (atom) | ECSA ($m^2$ $g^{-1}$) | MA (A $mg^{-1}$) | SA (mA $cm^{-2}$) |
|---|---|---|---|---|---|---|
| Pt/C | 20.05 | \ | \ | 111.6 | 0.34 | 0.30 |
| PtFe | 9.38 | 1.15 | 0.41 | 85.4 | 2.61 | 3.01 |
| PtCo | 9.83 | 1.25 | 0.52 | 54.2 | 2.10 | 3.87 |
| PtNi | 10.13 | 1.39 | 0.82 | 25.8 | 0.53 | 2.05 |

**Supplementary Table 3.** RDE ADT performance of the PtFe catalyst. ECSA was measured by CO stripping.

| | Initial | ADT-10,000 cycles | ADT-20,000 cycles | ADT-30,000 cycles |
|---|---|---|---|---|
| MA (A $mg^{-1}$) | 2.61 | 1.90 | 2.10 | 1.83 |
| SA (mA $cm^{-2}$) | 3.01 | 2.22 | 2.57 | 2.35 |
| ECSA ($m^2$ $g^{-2}$) | 86.71 | 85.73 | 81.65 | 77.79 |
| ECSA remain (%) | 100 | 98.8 | 94.2 | 90.0 |

**Supplementary Table 4.** RDE ADT performance of the Pt/C catalyst. ECSA was measured by CO stripping.

| | Initial | ADT-10,000 cycles | ADT-20,000 cycles | ADT-30,000 cycles |
|---|---|---|---|---|
| MA (A $mg^{-1}$) | 0.34 | 0.28 | 0.21 | 0.17 |
| SA (mA $cm^{-2}$) | 0.30 | 0.26 | 0.21 | 0.19 |
| ECSA ($m^2$ $g^{-2}$) | 111.6 | 106.9 | 98.6 | 87.8 |
| ECSA remain (%) | 100 | 95.8 | 88.3 | 78.7 |
